# Advances in stem cell-derived exosome therapy for radiation-induced skin injury

**DOI:** 10.1097/JS9.0000000000003004

**Published:** 2025-07-25

**Authors:** Pao-Jen Kuo, Cheng-Shyuan Rau, Ching-Hua Hsieh

**Affiliations:** a Department of Plastic Surgery, Kaohsiung Chang Gung Memorial Hospital and Chang Gung University College of Medicine, Kaohsiung, Taiwan; b Graduate Institute of Clinical Medical Sciences, College of Medicine, Chang Gung University, Taoyuan, Taiwan; c Department of Neurosurgery, Kaohsiung Chang Gung Memorial Hospital and Chang Gung University College of Medicine, Kaohsiung, Taiwan

**Keywords:** Radiation-induced skin injury (RISI), Stem Cell-Derived Exosomes, Therapeutic approach, Tissue regeneration

## Abstract

Radiation-induced skin injury (RISI) represents a significant clinical challenge, affecting up to 95% of radiotherapy patients and often resulting in both acute and chronic complications that can severely impact quality of life. While conventional treatments show limited efficacy, stem cell-derived exosomes have emerged as a promising cell-free therapeutic approach. These nanoscale vesicles, which harbor bioactive chemicals derived from their progenitor stem cells, exhibit significant potential in facilitating tissue healing while mitigating the risks associated with cell-based therapies. This article reviews the therapeutic processes of exosomes produced from stem cells in the treatment of RISI, including their roles in reducing cellular senescence, promoting angiogenesis, modulating inflammation, and enhancing tissue regeneration. We examine 12 preclinical data and five clinical trials, assessing the efficacy of exosomes sourced from several stem cell types: mesenchymal, embryonic, and induced pluripotent stem cells. The review also addresses current challenges in exosome therapy development, including manufacturing scalability, characterization standards, and delivery optimization. Recent advances in clinical translation suggest that exosome-based treatments could revolutionize RISI management, offering a safer and potentially more effective alternative to existing therapies. As the field progresses toward standardized therapeutic applications, stem cell-derived exosomes represent a paradigm shift in regenerative medicine approaches to radiation injury treatment.

## Introduction

Ionizing radiation can seriously harm the skin and produce acute and chronic injuries known together as radiation-induced skin injury (RISI)^[1]^. Radiotherapy causes some form of radiation dermatitis or skin damage in up to 95% of cancer patients^[2]^. Acute RISI presents as erythema, epilation, desquamation, or ulceration in the weeks following exposure, while chronic RISI can manifest months to years later as fibrosis, non-healing ulcers, or secondary malignancies^[3]^. RISI warrants a dedicated review due to its significant impact. Up to 95% of radiotherapy recipients are affected by this condition. It frequently interrupts cancer treatment and leaves lifelong sequelae. This creates an epidemiologic and clinical burden that surpasses most thermal or traumatic wounds^[2,3]^. Unlike burns or surgical injuries, irradiated skin exhibits persistent DNA damage, cellular senescence, and a chronic profibrotic inflammatory milieu that render conventional dressings, corticosteroids, and topical growth factors largely ineffective^[4,5]^. Moreover, the expanding population of cancer survivors is contributing to a global rise in acute and chronic RISI. The wider use of high-dose stereotactic protocols is also driving this increase. This trend underscores the urgent need for synthesizing emerging regenerative strategies. Stem cell-derived exosomes show particular promise as treatments tailored to this uniquely refractory wound type.

**HIGHLIGHTS**

- This review examines stem cell-derived exosomes for treating radiation-induced skin injury, offering a cell-free alternative to conventional therapies for up to 95% of radiotherapy patients.
- Exosomes facilitate wound healing through multiple mechanisms including reducing cellular senescence, promoting angiogenesis, modulating inflammation, and enhancing tissue regeneration with promising clinical trial results.
- Despite promising therapeutic potential, challenges remain in standardizing manufacturing processes, optimizing delivery methods, and establishing regulatory frameworks for clinical translation of exosome-based treatments.

Notably, RISI differs from typical wounds in its pathophysiology: irradiated skin exhibits impaired healing capacity, persistent inflammation, and a tendency for recurrent breakdown^[3]^. Ionizing radiation provokes clustered DNA double-strand breaks, persistent ROS overflow, endothelial apoptosis, and senescence-driven transforming growth factor beta 1 (TGF-β1) signaling – pathologies seldom observed in thermal or mechanical wounds^[2,3]^. These injuries severely affect patients’ quality of life and can necessitate interruptions in cancer treatment^[3,6]^. Unfortunately, few effective treatments exist to prevent or mitigate RISI aside from basic wound care; even advanced dressings, corticosteroids, or growth factors often fail to halt progression of radiation ulcers^[3,4]^. There is no standardized protocol for RISI management^[2]^, underscoring an urgent need for novel therapies.

Over the last two decades, regenerative approaches using stem cells have gained attention for treating radiation injuries^[3]^. Mesenchymal stem cells (MSCs) derived from bone marrow, adipose tissue, and other sources can migrate to injury sites, regulate inflammation, and facilitate tissue regeneration^[3]^. Preclinical studies showed that transplanting MSCs or stromal vascular fraction (SVF) can improve healing of irradiated skin wounds^[3]^. These benefits arise largely from paracrine effects: stem cells secrete growth factors, cytokines, and extracellular vesicles that stimulate repair of resident skin cells^[3,5]^. Among these secreted factors, exosomes have been identified as critical mediators of the therapeutic effects of stem cells^[5]^.

Exosomes are nano-vesicles (30–150 nm) of endosomal origin, secreted by virtually all cell types^[7]^. They carry diverse biomolecules including proteins, lipids, mRNAs, and microRNAs, protected by a lipid bilayer membrane^[7,8]^. This cargo can be delivered to target cells, altering gene expression and cell behavior in the recipient^[7,8]^. Notably, exosomes from stem cells recapitulate many of the pro-regenerative functions of their parent cells. These functions include promoting angiogenesis, dampening inflammation, and enhancing matrix remodeling. Importantly, exosomes provide these benefits without the risks associated with injecting live cells^[9]^. Because exosomes are biologically active yet replication-incompetent; they do not form tumors and elicit minimal immune rejection, making them a more secure alternative to whole-cell therapy^[9,10]^. Indeed, exosomes are endogenous to the body and typically do not trigger immune responses when administered^[5]^.

Recent studies indicate that exosomes produced from stem cells can facilitate the regeneration of irradiated skin, expediting wound healing and enhancing tissue quality^[3,5]^. Exosomes originating from many stem cell types, including MSCs, embryonic stem cells (ESCs), and induced pluripotent stem cells (iPSCs), have been evaluated for their therapeutic efficacy in skin regeneration. Each exosome source may carry a unique profile of regenerative signals. For example, exosomes from adult MSCs are enriched in anti-inflammatory and pro-angiogenic factors^[5,11,12]^, while exosomes from pluripotent stem cells carry developmentally related microRNAs that can rejuvenate senescent cells^[5,13,14]^. Harnessing these vesicles offers a cell-free strategy to treat RISI, circumventing concerns of graft-vs-host disease, immunogenicity, or tumorigenicity associated with direct stem cell transplantation^[3,9]^.

Adipose-derived stem cell (ADSC) exosomes retain the regenerative effects of ADSCs – such as promoting angiogenesis and modulating inflammation – while avoiding challenges of live cell therapies. They offer key clinical advantages over ADSC therapy, including lower risk of immune rejection and tumorigenicity, easier storage and transport, and better integration into biomaterials like hydrogels for targeted delivery^[15,16]^. Therefore, in this review, we examine how stem cell-derived exosomes exert therapeutic effects in radiation-injured skin. We first detail the mechanisms of action at the molecular level, highlighting key pathways modulated by exosomal cargo. We then summarize preclinical evidence from animal models demonstrating improved healing of irradiated wounds with exosome therapy. Next, we discuss current and emerging clinical applications, including ongoing trials of exosome-based treatments for skin injuries. Finally, we address challenges and future directions for translating stem cell-derived exosomes into clinical use for RISI.

### Search strategy and study selection

Following Preferred Reporting Items for Systematic Reviews and Meta-Analyses (PRISMA) 2020 recommendations^[17]^, we conducted a systematic literature search in PubMed/MEDLINE, Scopus, Web of Science, and ClinicalTrials.gov from database inception to 1 April 2025 using the Boolean string (“radiation-induced skin injury” OR “radiation ulcer” OR “radiodermatitis”) AND (“exosome” OR “extracellular vesicle” OR “MSC” OR “ADSC” OR “stem cell”). Two authors independently screened titles/abstracts, removed duplicates, and applied prespecified criteria (original data, English language, mammalian models, exosome isolation characterized per MISEV2018, and excluding duplicates, conference abstracts, and non-interventional studies), resolving discrepancies by consensus. A total of 950 records were identified across the databases from the search query, and 12 preclinical studies met the inclusion criteria. These consisted of *in vivo* animal experiments and *in vitro* cell studies demonstrating the effects of MSCs or MSC exosomes on radiation-induced skin injury. Five clinical studies met the inclusion criteria. These included human trials or case reports in which stem cell therapies were applied to patients with radiation-induced skin injuries. The main text and tables now note the study supporting each mechanistic or therapeutic statement of our literature coverage.

## Mechanisms of action of stem cell-derived exosomes in skin repair

**Figure 1.**
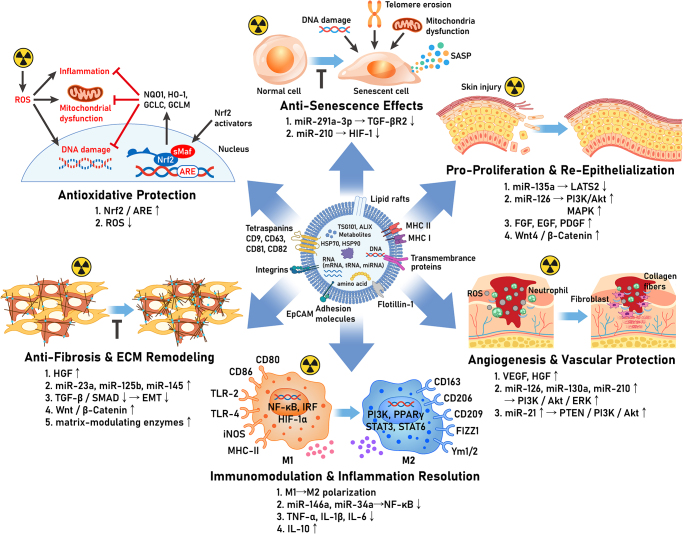
The schematic illustrates the multifaceted protective mechanisms and therapeutic pathways involved in radiation countermeasure strategies. The diagram centers around a bioactive intervention (likely extracellular vesicles or exosomes) containing various bioactive molecules including tetraspanins, integrins, adhesion molecules, lipid rafts, transmembrane proteins, and RNA species that collectively mediate radioprotective effects. Following ionizing radiation exposure (indicated by radiation symbols), the intervention activates six major protective pathways^[1]^: Antioxidative protection through Nrf2/ARE signaling, which upregulates antioxidant responses and reduces reactive oxygen species (ROS) while mitigating DNA damage and mitochondrial dysfunction^[2]^; anti-senescence effects via miR-291a-3p and miR-210 targeting TCF-βR2 and HIF-1α respectively, preventing cellular senescence and promoting tissue regeneration^[3]^; pro-proliferation and re-epithelialization mechanisms involving miR-135a, miR-126, growth factors (FGF, EGF, PDGF), and Wnt/β-catenin signaling to restore tissue integrity^[4]^; anti-fibrosis and extracellular matrix remodeling through HGF, microRNA regulation, and TGF-β/SMAD pathway modulation^[5]^; angiogenesis and vascular protection mediated by VEGF, HGF, and specific microRNAs targeting PI3K/Akt/ERK pathways^[6]^; and immunomodulation and inflammation resolution through M1-to-M2 macrophage polarization, microRNA-mediated NF-κB suppression, and cytokine balance restoration. This integrated approach demonstrates how targeted therapeutic interventions can simultaneously address multiple radiation-induced pathological processes to enhance survival and tissue recovery.

Stem cell-derived exosomes promote the healing of radiation-damaged skin through a multifaceted modulation of cellular processes. The key mechanistic pathways mediated by exosomes are summarized below (Fig. 1).

- **Inhibition of cellular senescence and DNA damage**: Radiation induces persistent DNA damage and premature senescence in skin cells, impairing their proliferative capacity^[4]^. Exosomes from stem cells carry specific microRNAs that counteract these effects. For example, ESC exosomes deliver miR-291a-3p, which targets TGF-β receptor 2 and thereby suppresses TGF-β signaling that drives cellular senescence^[18]^. In a study of irradiated human dermal fibroblasts, mmu-miR-291a-3p in ESC exosomes significantly reduced markers of senescence and accelerated closure of skin excisional wounds in aged mice^[18]^. Moreover, exosomal microRNA-210 has demonstrated the ability to enhance DNA repair in hypoxic environments through the modulation of hypoxia-inducible factor-1 signaling^[2]^. This can enhance the resolution of radiation-induced DNA breaks, improving cell survival. By delivering such nucleic acids, stem cell exosomes essentially “rejuvenate” radiation-damaged cells, pushing them back into the cell cycle for active repair^[5]^.
- **Promotion of keratinocyte proliferation and re-epithelialization**: Successful wound healing requires re-epithelialization – the migration and proliferation of keratinocytes to restore the epidermal barrier. Exosomal cargo from stem cells can accelerate this process. MSC exosomes are particularly rich in microRNAs that promote epithelial cell migration and proliferation. Notably, miR-135a contained in human amnion MSC exosomes inhibits the Hippo pathway kinase LATS2 in recipient cells^[19]^. LATS2 suppression leads to activation of pro-proliferative yes-associated protein (YAP)/ transcriptional co-activator with PDZ-binding motif (TAZ) signaling, thereby enhancing keratinocyte and fibroblast migration^[19]^. Exosomal miR-126 similarly promotes the phosphatidylinositol 3-kinase (PI3K)/Akt and MAPK pathways in skin cells, which are essential for cell survival and proliferation^[20]^. In a diabetic wound model, dressings loaded with miR-126-overexpressing MSC exosomes significantly improved epithelial coverage of the wound, likely via sustained PI3K/Akt signaling in keratinocytes^[20]^. Through these molecular routes, stem cell exosomes boost the regenerative capacity of the skin’s epithelial layer.
- **Angiogenesis and vascular protection**: Radiation damage to the dermal microvasculature contributes to hypoxia and delayed wound healing. Exosomes from stem cells help restore blood supply by promoting angiogenesis and protecting endothelial cells. MSC exosomes contain pro-angiogenic factors, including vascular endothelial growth factor (VEGF) and angiogenin, and microRNAs (e.g. miR-126 and miR-210) that stimulate new vessel formation^[2,7]^. Exosomal miR-126, for instance, is known to enhance the PI3K/Akt and extracellular signal-regulated kinase (ERK) pathways, which drive endothelial cell proliferation and migration^[20]^. In a study of ischemic wounds, sustained release of miR-126-rich exosomes led to significantly increased capillary density in the healing tissue^[20]^. Moreover, exosomes protect existing blood vessels from radiation injury. Endothelial cells exposed to radiation often undergo apoptosis due to oxidative stress and loss of survival signals. Exosomal microRNA-21 directly targets the phosphatase and tensin homolog (PTEN) gene in endothelial cells, activating the PI3K/Akt pathway and inhibiting apoptosis^[2]^. Overall, stem cell exosomes create a more favorable vascular environment in the wound bed: they encourage the growth of new microvessels and shield the endothelium from radiation-induced cell death.
- **Modulation of inflammation and immune response**: RISI features dysregulated inflammation, with excessive acute responses and chronic ulcers stuck in a pro-inflammatory state^[4,5]^. Stem cell exosomes modulate this inflammatory environment by acting on immune cells, particularly by shifting macrophages from pro-inflammatory M1 to pro-healing M2 phenotypes[6]. When inflammatory-stimulated, MSCs release exosomes enriched in miR-146a and miR-34a^[21,22]^. These microRNAs suppress inflammation through the nuclear factor kappa B (NF-κB) pathway and notch1 targeting, respectively, while promoting tissue remodeling genes [6]. Beyond macrophages, exosomes influence other immune cells to reduce inflammation and accelerate wound healing transition [18]. This is evidenced by human ADSC exosomes suppressing T-cell activity and inflammatory cytokine production [6], ultimately facilitating repair of irradiated skin.
- **Anti-fibrosis and remodeling of extracellular matrix**: Chronic radiation injuries feature excessive fibrosis and poor tissue repair. MSC exosomes help normalize this pathological process through targeted interventions. They inhibit TGF-β/ SMA and MAD homolog (SMAD) signaling and epithelial-mesenchymal transition processes. This inhibition reduces myofibroblast activation and prevents collagen overproduction [6]. Exosomal cargo may include antifibrotic microRNAs that downregulate fibrotic genes. In a murine radiation injury model, exosome therapy maintained the integrity of epidermal structures and decreased dermal scarring^[23]^. Additionally, bone marrow MSC exosomes activate Wnt signaling in endogenous stem cells, enhancing regeneration and reducing fibrosis^[24,25]^. Exosomes also modulate ECM turnover through matrix-modulating enzymes, resulting in repaired skin with improved collagen alignment and elasticity rather than rigid scarring^[26,27]^.
- **Protection against oxidative stress**: Ionizing radiation generates reactive oxygen species (ROS) in skin cells, leading to oxidative damage of proteins, lipids, and DNA that impairs healing^[28]^. Stem cell exosomes bolster the antioxidant defenses of irradiated skin. They have been found to activate the nuclear factor erythroid 2-related factor 2 (Nrf2) pathway, which is the master regulator of cellular antioxidant response^[5]^. In one study, MSC exosomes protected human keratinocytes from peroxide-induced oxidative injury by inducing Nrf2-dependent expression of antioxidant enzymes^[5,29]^. When Nrf2 was experimentally knocked down, the protective effect of the exosomes was lost. This finding confirms that exosomal factors rely on Nrf2 signaling to confer resistance to oxidative stress^[29]^. This is particularly relevant in RISI, where persistent ROS can create a vicious cycle of cellular senescence and chronic inflammation^[4]^. By upregulating Nrf2 and downstream detoxifying enzymes, exosomes help break this cycle, which accelerated healing of irradiated wounds by reducing lipid peroxidation, DNA oxidation, and ferroptosis (iron-dependent cell death) in the wound area, an effect attributed to enhanced expression of radiation-resistance and antioxidant genes in the tissue^[5,29]^. Thus, exosomal therapy fortifies the skin’s defenses against the oxidative onslaught of radiation.
- **Growth-factor priming of MSC exosomes**: Specific cytokines reshape exosomal cargo and influence their function. BMP-2 stimulation drives bone marrow-derived MSC (BMSC)/ADSC exosomes to accumulate miR-218, RUNX2 mRNA, and other osteo-inductive cues, markedly accelerating bone and matrix regeneration compared with naïve vesicles^[30]^. Immobilizing an FGF-2-derived peptide on culture plastic boosts Wharton’s jelly MSC exosome yield four-fold and loads them with proliferative proteins, offering a cost-efficient route to produce wound-healing vesicles at scale^[31]^. Hypoxia-induced VEGF signaling enriches vesicles in miR-205-5p and ANGPTL4, translating into superior endothelial tube formation and capillary density – attributes critical for re-vascularising irradiated dermis^[32]^. By contrast, TGF-β1 priming skews cargo toward miR-21-5p and CCN2, heightening fibroblast activation and scar formation, underscoring the need for stimulus-specific quality control^[33]^.

These synergistic mechanisms – anti-senescence, pro-proliferation, angiogenesis, immunomodulation, anti-fibrosis, and antioxidative effects – underlie the potent therapeutic action of stem cell-derived exosomes in RISI. It is important to note that the effects of exosomes are highly interconnected. For example, reducing inflammation via NF-κB inhibition also indirectly curtails fibrosis and improves regenerative signaling. The concerted action of exosomal molecules ultimately shifts the wound microenvironment from chronic damage to healing and regeneration.

## Preclinical evidence for exosome therapy in radiation skin injury

Extensive preclinical studies in cell culture and animal models have demonstrated the efficacy of stem cell-derived exosomes in treating radiation-induced skin injuries. Table 1 highlights key experimental and clinical studies. Below we summarize important contributions primarily from within the past five years:

- **Reversal of radiation senescence in fibroblasts (ESC exosome study)**: Bae *et al* provided early proof that exosomes from pluripotent stem cells can rejuvenate radiated skin cells^[18]^. They isolated exosomal miR-291a-3p from mouse ESCs and treated irradiated human dermal fibroblasts *in vitro*. This intervention markedly reduced senescence-associated β-galactosidase activity and p16^INK4a^ expression in the fibroblasts, indicating reversal of radiation-induced senescence^[18]^. *In vivo*, wounds in older mice (which heal slowly due to cellular senescence) healed significantly faster when treated with the ESC exosomes carrying miR-291a-3p than controls^[18]^. This study demonstrated a concrete mechanism by which exosomes can rescue radiation-damaged cells. The mechanism involves TGF-βR2 inhibition by miR-291a-3p. By targeting this pathway, exosomes restore the proliferative potential of cells damaged by radiation. This cellular recovery translates to improved wound healing^[18]^.
- **Enhanced wound healing via MSC exosomal microRNA (MSC exosome study**): Gao *et al* investigated human amnion MSC exosomes in a rat skin wound model^[19]^. They found these exosomes contained high levels of miR-135a, which we discussed above for its role in targeting LATS2. Rats with full-thickness skin wounds were treated with topical MSC exosomes. The treated wounds showed significantly accelerated closure, with histological evidence of enhanced re-epithelialization and collagen organization compared to untreated wounds^[19]^. Mechanistically, exosome treatment enhanced two critical healing processes. Keratinocyte migration increased at wound edges, and granulation tissue formation improved. These effects are consistent with miR-135a promigratory action in skin cells^[19]^. While this study was in non-irradiated wounds, it provides strong evidence of MSC exosomes’ ability to expedite healing and carries the potential to be applied to radiation injuries as well.
- **Protection against oxidative skin injury by MSC exosomes (radiation oxidative stress study**): Wang *et al* explored how MSC exosomes mitigate oxidative damage in skin, which is directly relevant to radiation exposure^[29]^. Using a combination of *in vitro* and *in vivo* models, they showed that exosomes from human MSCs activate the Nrf2 pathway in recipient skin cells^[29]^. In cultured human keratinocytes subjected to ionizing radiation or hydrogen peroxide, addition of MSC exosomes led to upregulation of Nrf2 and its downstream antioxidant enzymes (like heme oxygenase-1 and superoxide dismutase)^[5,29]^. This resulted in lower intracellular ROS levels and higher survival of the keratinocytes compared to controls. Similarly, in a mouse model of radiation dermatitis, topical application of MSC exosomes significantly reduced epidermal oxidative damage and led to faster resolution of lesions^[29]^. Importantly, blocking Nrf2 in these models abolished the protective effect, confirming the mechanism. These findings demonstrate that MSC exosomes can guard the skin against radiation-induced oxidative stress, which is a major contributor to tissue injury.
- **Immunomodulation and bystander effect mitigation (inflammation studies**): Research indicates that exosomes from inflammatory-stimulated adipose-derived stem cells (ADSCs) show improved immunosuppressive effects by raising anti-inflammatory interleukin-10 (IL-10) and lowering pro-inflammatory cytokine activity^[34]^. These findings suggest potential applications for chronic radiation wounds. Research also reveals that irradiated cells release exosomes with altered microRNAs that cause DNA damage and senescence in non-irradiated cells through bystander effects^[2,5]^. Introducing healthy MSC exosomes may counteract these damaging signals, though this remains under investigation. This exosome-based cellular communication presents a promising approach to mitigate radiation bystander damage.
- **Healing of irradiated wounds in animal models**: *In vivo* studies demonstrate stem cell exosomes’ efficacy in RISI models. Gan *et al* showed that plasma-derived exosomes accelerated wound healing in irradiated mouse skin, improving closure and re-epithelialization while reducing ferroptosis through enhanced radioprotective gene expression^[23]^. Similarly, Bai *et al* found that extracellular vesicles from irradiated neonatal mouse tissue prevented radiation-induced hair loss by preserving follicular stem cells and enhancing DNA repair^[7]^. These findings demonstrate stem cell-derived vesicles’ capability to protect and restore irradiated skin structures.
- **Stem cell secretome for chronic radiation ulcers**: A recent advance is the use of complete stem cell secretome rich in exosomes to treat chronic radiation ulcers. Chen *et al* developed a chronic radiation ulcer model in rats by locally irradiating skin and observed progressive, non-healing ulceration with features of cellular senescence^[4]^. The treatment protocol involved repeated injections of a specialized medium. This conditioned medium was derived from human umbilical cord MSCs and contains mostly exosomes. The goal was to heal the radiation-induced ulcers^[4]^. Strikingly, the MSC-conditioned medium (MSC-CM) significantly slowed the progression of the radiation ulcers and improved healing outcomes compared to vehicle controls^[4]^. Histologically, MSC-CM-treated ulcers had fewer senescent cells and reduced expression of senescence markers like p16^INK4a^^[4]^. The treatment also dampened chronic inflammatory cell infiltration and promoted re-epithelialization at the ulcer margins. The authors attributed these benefits largely to the exosomes and soluble factors in the MSC secretome that inhibited the senescence-associated secretory phenotype (SASP) in the wounds^[4]^. This study provides a direct proof-of-concept that stem cell-derived exosomes/secretions can effectively treat refractory radiation ulcers. It mirrors earlier reports where MSC or SVF cell grafts mitigated radiation injuries^[3]^, but importantly achieves the result with a cell-free approach.

**Table 1:** Key clinical trials and studies on stem cell-derived exosomes for skin injury (including radiation-induced injuries and related wound healing applications)

Study/Trial (year)	Condition (injury type)	Exosome source (cell type)	Delivery method	Outcome/Status
Rion “RioDerm-003” Phase I (ongoing, 2024)^[37]^	Radiodermatitis (prevention in cancer radiotherapy)	Platelet-derived exosomes (purified exosome product, PEP)	Topical powder in hydrogel (applied to irradiated skin)	Phase I in Progress; no immune reactions observed to date; assessing safety/tolerability in radiation dermatitis patients
Traub *et al* 2021 (case report)^[6]^	Radiation dermatitis (chronic ulcer post-radiotherapy)	Mixed skin stem cell secretome (S2RM technology; includes MSC and dermal fibroblast exosomes)	Topical gel, applied daily to wound	Complete healing of radiotherapy-induced skin lesion in 4 weeks; marked reduction in pain and inflammation
Park *et al* 2023 (RCT)^[38]^	Laser-induced skin injury (ablative fractional laser for acne scars)	Adipose-derived MSC exosomes (commercial cosmetic product)	Topical, post-laser (split-face study)	Significantly improved scar outcomes vs. control; ~13% greater wrinkle reduction; higher skin elasticity; reduced post-procedure redness
Rion PEP Phase II (DFU) 2023^[35,37]^	Chronic diabetic foot ulcers (non-radiation chronic wounds)	Platelet-derived exosomes (PEP)	Topical gel (exosomes mixed in fibrin sealant)	Completed Phase II; preliminary reports indicate improved wound closure rates; potentially informative for radiation ulcer approaches
NCT05475418 (recruiting, 2022)^[35,39]^	Chronic cutaneous ulcers (mixed etiologies)	ADSC exosomes	Topical exosome dressing + hydrogel	Recruiting; evaluating safety and efficacy; outcomes include percent wound area reduction and healing time
Chen et al. 2023 (preclinical rat study)^[4]^	Chronic radiation ulcer (rat model)	Umbilical cord MSC secretome (exosome-rich CM)	Intraperitoneal injections (multiple doses)	Significantly slower ulcer progression and enhanced healing; provided rationale for human translation

Abbreviations: RCT, randomized controlled trial; MSC, mesenchymal stem cell; DFU, diabetic foot ulcer; CM, conditioned medium.

These preclinical findings demonstrate the strong therapeutic potential of stem cell-derived exosomes for radiation-induced skin damage. The benefits of exosome treatments have been consistently demonstrated across multiple experimental approaches. Studies have ranged from cellular assays to complex rodent wound models. Across all these models, exosome treatments produced superior outcomes relative to controls. These benefits include faster wound closure, reduced inflammation and fibrosis, enhanced angiogenesis, and improved tissue regeneration^[5,35]^. The mechanisms identified are supported by direct observations of molecular changes in treated wounds (e.g. increased proliferation markers, higher angiogenic factor levels, and lower oxidative stress markers)^[5]^. The therapeutic benefits of exosomes extend across multiple stem cell sources. Researchers have observed similar positive effects from adult MSCs derived from the bone marrow, adipose tissue, and amnion. ESCs and iPSC-derived cells also produce beneficial exosomes. While each source’s exosomes may excel in particular aspects, all contribute meaningfully to the overall repair process.

## Clinical applications and translational studies

The translation of stem cell-derived exosome treatment from laboratory to clinical application for skin lesions is a dynamic and promising field of research. Despite the current limitations in clinical experience, preliminary investigations have indicated the safety and suggested the efficacy of exosome-based therapies in humans^[5,36]^. Importantly, multiple clinical trials are underway to evaluate exosome therapy in various skin injury contexts, including radiation dermatitis. Here, we summarize the current state of clinical and translational efforts in Table 1.

### Safety and feasibility

Initial trials have focused on establishing the safety profile of exosome products in humans. Exosomes are generally well-tolerated; being acellular, they evade many immune detection mechanisms^[5]^. For instance, cancer patients receiving exosome-based immunotherapies have shown no serious adverse immune reactions^[5]^. In dermatology, a 2020 randomized trial by Park *et al* used topical ADSC exosomes to treat facial skin after laser resurfacing and reported no significant side effects, only mild and transient redness similar to placebo^[38]^. These findings support that exosome therapies can be safely administered (topically or by injection) to human skin. Dosage-ranging studies are still needed, but thus far, doses on the order of 10^9–10^11 exosome particles per mL appear safe and biologically active^[35]^.

### Treatment of radiation dermatitis

One exciting application is using exosomes to mitigate acute radiation dermatitis during cancer radiotherapy. A proprietary exosome-based product known as RioDerm-003 (developed by Rion Therapeutics) is currently in a Phase I clinical trial for radiodermatitis in patients^[37]^. RioDerm-003 consists of purified platelet-derived exosomes in a topical carbomer hydrogel formulation^[37]^. The choice of platelet exosomes is based on their natural role in wound healing. This product is applied as a powder or gel to radiation-exposed skin to prevent or reduce radiation dermatitis. While results are pending, the advancement of RioDerm-003 into clinical testing underscores translational progress – moving from concept to a regulated therapeutic candidate. In parallel, researchers are exploring when to apply exosome therapy for best results (prophylactically during radiation vs. reactively after injury). Preclinical data suggest applying exosomes during the course of radiation can protect normal skin without protecting tumor cells if delivered locally^[5]^. However, careful clinical studies are needed to ensure that using radioprotective exosomes on a cancer patient’s skin does not inadvertently shield the tumor. Approaches like localized topical delivery help confine the effect to skin.

### Chronic wounds and ulcers

Chronic cutaneous radiation ulcers (e.g. from prior radiotherapy or accidental exposure) are an area of great clinical need. As described earlier, a case report by Traub *et al* documented successful treatment of a radiation dermatitis wound using a topical stem cell-derived secretome product^[6]^. In that case, a breast cancer patient with a persistent radiotherapy-induced skin lesion was treated with a gel containing molecules and exosomes from multiple skin stem cell types (S2RM technology). The patient experienced rapid pain relief, reduced redness, and complete healing of the lesion within weeks, with restoration of normal skin appearance^[6]^. Although this is a single-patient report, it provides a real-world example of using a cell-free regenerative therapy for radiation skin damage. Building on such successes, more formal trials are planned. One Phase I trial NCT04128126 aims to use CM from Wharton’s jelly MSCs (rich in exosomes) to treat chronic radiation ulcers and assess wound closure rates^[35]^.

Additionally, knowledge from exosome use in non-radiation chronic wounds is feeding into RISI management. Diabetic foot ulcers (DFUs) are chronic wounds that share features of impaired healing with radiation ulcers. In 2023, Rion Therapeutics completed a Phase II trial of their purified exosome product (PEP) in DFU patients^[35]^. In that study, patients received a fibrin sealant combined with PEP topically to the ulcer. While detailed results are not yet published, the trial’s progression to Phase II indicates promising early outcomes. Another ongoing trial in China (NCT05475418) is testing ADSC exosome dressings in combination with a hydrogel for chronic skin ulcers^[40]^. These studies, while not specific to radiation, are directly informative: a positive result in DFUs would support using similar exosome therapies for chronic radiation wounds. Indeed, the mechanisms of action (enhancing angiogenesis, re-epithelialization, etc.) are shared across different wound etiologies.

### Aesthetic and dermatologic procedures

Beyond therapeutic injury repair, exosomes have entered cosmetic dermatology practice, which provides additional evidence of their efficacy in human skin. For example, Park *et al* performed a split-face randomized trial on patients with atrophic acne scars^[38]^. One side of each patient’s face was treated with fractional CO_2_ laser plus topical ADSC exosomes, while the other side received the laser plus a placebo. The exosome-treated sides showed significantly greater scar improvement, higher collagen density, and reduced post-laser erythema compared to control sides. This 12-week study demonstrated in a controlled setting that stem cell exosomes can enhance skin regeneration and remodel acne scar tissue in humans^[40]^. Similarly, exosome-based products are being used experimentally to promote hair regrowth after chemotherapy or radiation-induced alopecia, given the encouraging preclinical results on hair follicle repair^[7]^. These esthetic applications are tangential to medical wound healing. However, they reinforce an important concept about exosome function. They demonstrate that exosomes are active in human skin repair and can be delivered topically with benefit.

### Route of delivery and formulation

Clinical translation has spurred innovations in how exosomes are delivered to patients. Topical application on the skin as a liquid, cream, or hydrogel dressing is non-invasive and has been used in most skin trials to date^[35,37]^. For deeper or more chronic lesions, intradermal or subcutaneous injections around the wound margin are also explored^[4]^. To improve retention of exosomes at the wound site, biomaterials such as hydrogels are being employed. A thermosensitive hydrogel can be mixed with exosomes to form a depot that slowly releases vesicles over time^[2]^. This prolongs the interaction of exosomes with the injured tissue, potentially enhancing efficacy. One trial is testing a sprayable hydrogel scaffold loaded with exosomes for this reason (NCT05593311, for wound healing). Another innovation is lyophilized or powdered exosome formulations (like RioDerm-003^[37]^), which offer better stability and ease of use. These advances in formulation are crucial for moving exosome therapy into routine clinical use, where shelf-life, sterility, and ease of application become important considerations.

Collectively, clinical research on stem cell-derived exosomes for skin repair is advancing at a rapid pace. It is evident from preliminary research that these remedies are safe and can enhance the healing process in a variety of skin injuries. For RISI specifically, ongoing trials will determine the degree of benefit in both preventive (mitigating radiotherapy dermatitis) and therapeutic (healing chronic ulcers) settings. The path to regulatory approval will require robust data from controlled studies, but the trajectory is encouraging. As more is learned, we anticipate exosome therapy could become an integral part of managing RISI.

## Comparative effectiveness of exosomes from different stem cell sources

All stem cell-derived exosomes share a core ability to promote tissue repair, but there are nuanced differences in their cargo composition and therapeutic profiles depending on the cell source. Table 2 examines the characteristics and efficiency of exosomes derived from three primary stem cell types: MSCs, ESCs, and iPSCs, in the context of skin injury treatment. Notably, exosome source fundamentally dictates cargo profiles and, by extension, their wound-healing mechanisms. Comparative profiling confirms that MSC exosomes versus ESC or iPSC exosomes carry distinct microRNA and protein payloads, engaging different healing pathways^[41,42]^. MSC exosomes are enriched in anti-inflammatory, pro-angiogenic microRNAs (e.g. miR-21-5p and miR-146a-5p) that modulate key signaling cascades to foster repair^[41]^. For example, exosomal miR-146a-5p targets TRAF6 in the NF-κB pathway, skewing macrophages toward an anti-inflammatory M2 phenotype and accelerating re-epithelialization^[43]^. Likewise, MSC exosomal miR-21 can fine-tune TGF-β/Smad signaling in dermal fibroblasts – curbing myofibroblast activation and excessive scarring^[44]^. In contrast, ESC exosomes or iPSC exosomes harbor abundant pluripotency-associated miRNAs (such as the miR-302/372 family) alongside growth factors that activate regenerative pathways. Notably, human iPSC exosomes contain FGF2, which engages FGFR3/p38 MAPK signaling in keratinocytes to spur proliferation and migration^[45]^. Pluripotent cell exosomes are also rich in effectors that stimulate Wnt/β-catenin and PI3K/Akt pathways, thereby promoting robust re-epithelialization and cell survival^[46]^. Furthermore, MSC exosomal cargo can activate cytoprotective programs like Nrf2; indeed, exosomes from Nrf2-overexpressing MSCs boost angiogenesis and dampen oxidative stress in diabetic wounds, markedly improving healing outcomes^[47]^. These cell type-specific cargo differences translate into functional specializations: ESC/iPSC exosomes tend to exert stronger direct pro-regenerative effects (driving proliferation, angiogenesis, and tissue regeneration via Wnt, Akt, etc.), whereas MSC exosomes excel at immunomodulation and inflammation resolution^[41]^. In sum, the heterogeneity in exosomal payload (microRNAs, proteins, and signaling molecules) underlies divergent intercellular communication profiles – with each exosome type converging on overlapping yet distinct molecular targets (TGF-β, PI3K/Akt, Wnt/β-catenin, NF-κB, Nrf2, among others) to synergistically promote cutaneous wound healing^[41]^.

**Table 2:** Comparative profile of stem cell-derived exosomes for skin injury therapy

Stem cell source	Key beneficial features of exosomes	Limitations/Considerations	Representative references
Mesenchymal stem cells (MSCs)	Anti-inflammatory properties promoting M2 macrophages Inhibit fibrosis and irregular collagen deposition Enhance angiogenesis via miR-126 and growth factors Improve re-pithelialization through miR-135a, Wnt4 Low immunogenicity for allogeneic use Extensive preclinical evidence and ongoing clinical trials	Donor variability affecting exosome content Limited anti-senescence miRNAs vs. pluripotent sources Scalability challenges for clinical-grade production	MSC exosome content and therapeutic functions^[9]^
			Domenis 2018 – immunosuppressive function^[34]^
Adult sources: e.g. bone marrow, adipose, umbilical cord			
			Wang 2020 – Nrf2 oxidative protection^[29]^
			Gan 2021 – irradiated wound healing^[23]^
			Rion trials – multiple in Table 1 ^[37]^
Embryonic stem cells (ESCs) Pluripotent stem cells from blastocyst	Strong anti-senescence activity through miR-290 family Enhanced wound closure in challenging contexts Rich in developmental morphogens aiding tissue regeneration High potency per particle potentially requiring lower doses	Ethical and regulatory constraints limiting clinical translation Allogeneic nature potentially triggering immunogenicity Concerns about tumor-supporting factors (telomerase) Limited clinical trials compared to MSC-derived products	Bae 2019 – ESC exosomal miR-291a reversing fibroblast senescence^[18]^
			Khan 2015 – ESC exosomes in cardiac repair^[48]^
			Yang 2023 – notes ESC contributions in RISI repair^[3]^
Induced pluripotent stem cells (iPSCs)	Combines pluripotent capacity with autologous potential Unlimited supply from renewable source Customizable through lineage-specific differentiation Amenable to genetic engineering (miR-126, miR-146a) Promising results in diabetic wounds and radiation-induced alopecia	Complex, costly manufacturing process Rigorous safety monitoring requirements Undefined regulatory pathway Limited clinical data despite preclinical success	Zhang 2015 – iPSC exosomes heal wounds comparably to MSC exosomes^[49]^
			Gupta 2024 – concept of iPSC for radiation injury^[50]^
Somatic cells reprogrammed to pluripotency			
			Chen 2023 – large-scale production of iPSCs-derived exosomes^[51]^

### MSC exosomes

MSCs (from bone marrow, adipose tissue, umbilical cord, etc.) are the most extensively studied source of therapeutic exosomes for wound healing. MSC exosomes are naturally equipped with immunomodulatory and pro-regenerative molecules reflective of MSCs’ role in supporting tissue repair^[9]^. They contain numerous growth factors, including TGF-β3, VEGF, and hepatocyte growth factor (HGF), anti-inflammatory cytokines (e.g. IL-10), and hundreds of microRNAs (e.g. miR-21, miR-146a, and miR-126) known to regulate healing processes^[21,52]^. As discussed, these exosomes can reduce inflammation, enhance angiogenesis, and prevent fibrosis effectively. Comparative studies suggest that exosomes from ADSC might have particular advantages in cutaneous repair – they strongly inhibit scar formation and abnormal collagen deposition^[2]^. ADSC exosomes have been noted to increase the collagen III/I ratio and elastin in healing skin, improving scar quality^[9]^. Bone marrow MSC exosomes similarly attenuate fibrosis and inflammation, as shown in models of lung and bone radiation injury^[5,26,53]^. A key benefit of MSC exosomes is their low immunogenicity: since they lack major cell surface proteins, they usually do not trigger immune rejection even when derived from an allogeneic donor^[5]^. This enables off-the-shelf use. One consideration, however, is variability – exosome content can vary based on the tissue source and donor conditions^[9]^. For example, exosomes from older donors or different tissue MSCs might be less potent. Standardizing MSC exosome production is an active area of research. Overall, MSC exosomes are a frontrunner for skin therapies due to their combination of efficacy and safety. Indeed, most current clinical trials utilize MSC or MSC exosomes.

### ESC exosomes

ESC exosomes contain unique developmental regulators, including the miR-290 family, which can reverse cellular senescence. Bae *et al* demonstrated that ESC exosomal miR-291a-3p reduces radiation-induced fibroblast senescence^[18]^. While ESC exosomes show superior proliferative stimulation compared to MSC exosomes due to factors like miR-302/367, their clinical application faces challenges^[54]^. These include ethical concerns, allogeneic nature, and potential oncogenic factors like LIN28 and telomerase components^[55]^. Although exosomes lack DNA and cannot directly induce uncontrolled growth, regulatory approval remains complex. Alternative approaches include using parthenogenetic ESCs or human leukocyte antigen (HLA)-matched banking for universal donors. Despite powerful regenerative potential, ESC exosomes’ clinical use is currently limited by ethical and immunological considerations

### iPSC exosomes

Zhang *et al* demonstrated that iPSC exosomes accelerated wound healing compared to native MSC exosomes, improving collagen and elastin deposition^[49]^. iPSCs enable unlimited, potentially autologous exosome production, with proven non-immunogenic effects in pig wound models^[35]^. While current good manufacturing practice (GMP) is costly and requires careful safety monitoring, iPSC exosomes offer unique advantages: they can be genetically modified to enhance therapeutic factors, as shown in miR-126-enriched exosomes improving diabetic wound angiogenesis^[2,5]^. Despite promising preclinical results, widespread implementation awaits simplified production and safety validation. Future developments may enable mass-produced “off-the-shelf” iPSC exosomes for RISI treatment.

## Comparative limitations of stem cell-derived exosome sources

Some key limitations that differentiate ESC, iPSC, and MSC exosomes in a translational context should be recognized. Table 3 compares the limitation of exosomes isolated from various stem cell sources.

**Table 3:** Comparative limitations of stem cell-derived exosome sources

Exosome source	Principal limitation	Translational impact	Recent representative evidence	Potential mitigation
ESC exosomes	Ethical and regulatory controversy (embryo destruction) Potential oncogenic factors (e.g. LIN28, telomerase) allogeneic immunogenicity	Slows regulatory approval and may limit patient acceptance and reimbursement pathways	Khandia *et al*, 2024^[56]^	Parthenogenetic or HLA-banked ESC lines Stringent purification to deplete residual pluripotency proteins Robust informed-consent frameworks
			Ranjan *et al*, 2023^[54]^	
iPSC exosomes	Very limited human clinical data (only early-phase safety studies) Costly, complex GMP manufacturing Possible genomic-instability–related safety concerns	Uncertain benefit-risk profile and lack of precedents impede regulatory and payer confidence	NCT05886205, 2023^[57]^	Launch multicentre Phase I/II trials with harmonized endpoints Implement closed-system bioreactors and release-potency assays Exploit autologous or iMSC derivatives to reduce tumorigenic risk and costs
			Chen *et al*, 2023^[4]^	
			Kirkeby *et al*, 2025^[58]^	
MSC exosomes	High donor-to-donor variability and tissue-source heterogeneity alter exosome cargo and potency	Batch-to-batch inconsistency causes variable clinical outcomes and complicates product comparability	Česnik *et al*, 2024^[59]^	Establish well-characterized donor banks or clonal MSC lines Pool donors to average variability Deploy cargo-based potency assays and AI-driven QC Pre-conditioning to normalize vesicle profiles
			Jia *et al*, 2022^[60]^	
			Xie *et al*, 2021^[61]^	

### ESC exosomes

ESC exosomes possess unrivalled pro-proliferative potency, yet their derivation from human embryos raises profound ethical and legal questions, echoed by recent neuroregenerative-therapy reviews^[56]^ and retrodifferentiation of retinal Müller cells^[62]^. These concerns, combined with residual pluripotency proteins that could support tumor growth, currently cap their clinical trajectory. We therefore emphasise mitigation strategies – parthenogenetic or HLA-typed lines and deeper purification – to demonstrate a viable regulatory path.

### iPSC exosomes

iPSC exosomes circumvent embryo ethics but face a different bottleneck: scant human data. To date, only single-center, early-phase trials (e.g. NCT05886205 for refractory epilepsy) have entered the clinic, leaving efficacy claims largely pre-clinical^[57]^. Manufacturing at the GMP scale remains labour-intensive despite recent progress, and comprehensive safety monitoring is mandatory because reprogramming can introduce genomic aberrations. The 2025 pluripotent-product landscape review likewise notes that exosome products lag far behind cell-based hPSC therapies in trial numbers^[58]^. We outline concrete next steps – multisite Phase I/II studies, closed-system bioprocessing and use of iMSC derivatives – to accelerate translation.

### MSC exosomes

MSC exosomes enjoy the widest pre-clinical and early clinical use but are hindered by donor variability. Large cohort analyses show two-fold differences in proliferation and immunomodulatory capacity between umbilical-cord MSC donors^[59]^, and similar heterogeneity has been linked to inconsistent wound-healing endpoints^[60,61]^.

## Challenges and future directions

While stem cell-derived exosomes hold great promise for treating RISI, several challenges must be addressed as the field progresses toward widespread clinical adoption. Here, we discuss key hurdles and future directions.

### Manufacturing and scalability

Producing high-quality exosomes at scale is a major challenge. Each batch of exosomes must be isolated from cultured cells, requiring expansion of millions of stem cells. Manufacturing processes need to be standardized to yield reproducible exosome preparations with consistent potency^[9]^. Techniques like ultrafiltration, size-exclusion chromatography, and tangential flow filtration are being refined to purify exosomes efficiently for clinical use. Bioreactor-based cell culture systems can help scale up production by growing stem cells in large quantities. Additionally, lyophilization methods are being explored to create stable exosome powders (as with Rion’s product) for easier storage and transport^[37]^. Furthermore, the field is moving toward GMP compliant exosome production, which will be essential for regulatory approvals. Although large-scale exosome production remains a major barrier for exosome clinical applications, a few promising works have been presented. Hollow-fiber bioreactors now yield up to 1.8 × 10^10^ ADSC exosomes mL^−1^ – 38-fold more than flasks – delivering >10^13^ particles per 10-day GMP run^[63]^. Burn and radiation models demonstrate that spraying or hydrogel-loading 5 × 10^8^–10^10^ particles over 1–1.5 cm^2^ wounds (≈10^9^ cm^−2^) halves healing time and limits scarring^[64,65]^. Extrapolating these doses indicates a single bioreactor batch could treat ~ 100 cm^2^ skin defects, confirming ADSC exosomes are both scalable and clinically practical for large-area wounds.

### Characterization and potency assays

Alongside manufacturing, there is a need for rigorous characterization of exosome products. Exosomes are complex: they contain thousands of proteins and RNAs. Determining which components are critical for therapeutic effects is an ongoing area of research. Potency assays, which evaluate the therapeutic efficacy of a certain exosome batch, are essential for quality control. The International Society for Extracellular Vesicles (ISEV) has issued position papers regarding the minimal characterization of exosomes, encompassing markers such as CD63, CD81, and particle size analysis^[9]^. Future standards will likely require demonstrating particle identity, purity (minimal protein/lipid contaminants), and batch-to-batch consistency in cargo profiles before clinical use.

### Delivery methods and targeting

Getting exosomes to the site of injury in sufficient concentration is another challenge. For superficial skin injuries, direct topical application is straightforward. However, deeper wounds or chronic ulcers present retention challenges. In these cases, exosomes may wash away or diffuse before they can provide therapeutic benefit. To address this, advanced delivery systems are being developed. Hydrogels and wound dressings that slowly release exosomes have shown success in preclinical models^[2]^. For example, a chitosan hydrogel was used to deliver miR-126-enriched exosomes over several days, yielding improved healing in diabetic wounds^[2]^. Such sustained-release systems ensure a therapeutic level of exosomes is maintained at the wound. Another strategy involves targeting exosomes to specific cells. This can be achieved by engineering exosome surface proteins. For example, researchers can display an antibody or peptide that homes to wound endothelium. This approach would concentrate the therapeutic effect where it is needed most^[2]^. This field of exosome engineering is rapidly expanding, with techniques to load drugs or modify surface ligands on exosomes^[2,66,67]^. In the future, we might see “smart exosomes” that deliver cargo in response to the wound microenvironment or specifically seek out radiation-damaged cells.

### Emerging potential of exosome engineering

Rapid advances in exosome engineering now enable rational customization of vesicle surfaces, tropism, and cargo to overcome the hypoxic, fibrotic barriers that typify radiation-injured skin. Surface chemistries such as 1-ethyl-3-(3-dimethylaminopropyl)carbodiimide (EDC) and N-hydroxysuccinimide (NHS) coupling or click reactions, combined with 1,2-distearoyl-sn-glycero-3-phosphoethanolamine conjugated to polyethylene glycol (DSPE-PEG) insertion, let RGD peptides, epidermal growth factor receptor or CD44 aptamers be stably displayed on exosome membranes, doubling retention in integrin-rich wounds while prolonging circulation half-life^[68]^. Decorating vesicles with superparamagnetic iron-oxide nanoparticles yields “magnetosomes” that an external field can pull to the treatment site; a 2024 mouse study showed remotely guided MSC exosomes delivering doxorubicin with high precision – an easily translatable strategy for regenerative cargos in large skin defects^[69]^. Formulation engineering is equally powerful: an oxygen-nanobubble hydrogel coated with exosomes boosted intradermal uptake four-fold and sped closure of 2 cm^2^ diabetic wounds, illustrating how smart biomaterials provide area-wide, sustained release on hypoxic ulcer beds^[70]^. Therapeutic payloads are now programmable – electroporation, sonication, or EXO-code tags achieve >90 % encapsulation of mRNA or siRNA, with 2025 platforms demonstrating robust *in vivo* translation and gene-repair activity^[71]^. These strategies converge: surface-targeted, miRNA-enriched vesicles already deliver superior angiogenesis, anti-fibrosis, and scar attenuation in pre-clinical wound and radiation-ulcer models, underscoring their translational promise^[72]^. Together, surface modification for homing, magneto- or hydrogel-based delivery for uniform coverage, and tailored nucleic-acid or protein cargoes amplify therapeutic potency while keeping dosing economical, paving the way for next-generation, cell-free biologics in extensive cutaneous injuries.

### Timing and dosing strategies

Treatment regimens for exosome therapy in radiation injuries require optimization. Prophylactic application shows promise, with MSC exosomes limiting acute damage when given immediately post-radiation^[28]^. Radiotherapy patients could benefit from tailored exosome delivery strategies. One approach involves applying exosomes after each radiation fraction to reduce cumulative skin injury. Another strategy uses regular dosing schedules, such as weekly or biweekly treatments, specifically for chronic ulcers. Combination approaches might be effective: initial injections followed by exosome-infused dressings. However, the non-linear dose-response relationship necessitates careful optimization to maximize benefits while considering production costs.

### Regulatory and safety considerations

Exosome therapeutics represent a novel regulatory category distinct from cells and traditional drugs. While small clinical trials show no significant safety concerns^[5,36]^, challenges include potential tumor interactions in radiotherapy patients, necessitating localized rather than systemic application. Long-term effects are expected to be minimal due to exosomes’ transient nature and inability to replicate, making them potentially safer than cell therapies. Emerging regulatory frameworks will require manufacturers to demonstrate freedom from adventitious agents and harmful impurities.

### Cost and accessibility

Like many advanced biotherapies, cost could be a barrier initially. Producing exosomes involves cell culture and purification processes that are more expensive than synthesizing small-molecule drugs. However, the cost should be viewed in context with current treatment expenses. Treating a chronic radiation ulcer currently requires multiple surgeries or long-term wound care, which are also costly. If exosome therapy can significantly reduce healing time and complications, it may justify the initial cost. Over time, costs are expected to decrease as manufacturing is scaled up and optimized. This optimization could include automation or even synthetic exosome mimetics. Another cost-reduction approach involves using allogeneic exosome sources, such as an MSC bank, to produce bulk product. This would be more economical than custom-making exosomes for each patient. Many companies are pursuing centralized manufacturing for off-the-shelf use, which will improve accessibility.

### Research directions

Future research aims to deepen mechanistic understanding through “omics” analyses of exosome components. This knowledge guides enhancement strategies, including pre-conditioning donor cells (like hypoxia or inflammatory exposure) to modify exosome content^[4]^. For example, interferon-gamma-γ (IFN-γ) priming increases MSC exosomal miR-126-3p, improving angiogenesis in diabetic wounds^[73,74]^. Similar approaches could yield radiation-specific exosomes by pre-stressing cells with radiation. Future directions also include combination therapies, potentially synergizing exosomes with growth factors, cellular therapies, or standard wound care treatments.

In conclusion, the future of stem cell-derived exosome therapy necessitates addressing production and regulatory obstacles, optimizing transport and dosing, and ensuring cost-efficiency. The field is rapidly evolving, and lessons learned from the first generation of trials will inform improved second-generation products. There is optimism that many of these challenges are surmountable. Exosomes possess several practical advantages that many cell therapies lack. These include their intrinsic stability, ability to be stored, and relative ease of application. For example, they can be applied topically like a simple cream or gel^[9]^. As research continues, we anticipate that exosome-based treatments will become more standardized, with defined composition and robust clinical data.

## Conclusion

Stem cell-derived exosomes have emerged as a novel and powerful modality for treating radiation-induced skin injuries. By concentrating the healing signals of stem cells into tiny lipid vesicles, exosome therapy provides a cell-free approach that addresses the key pathological features of RISI: impaired cellular function, dysregulated inflammation, microvascular damage, and fibrosis. As we move closer to realizing this vision, we enter a new era of “vesicle therapeutics” for radiation injuries. These nanoscale messengers of healing encapsulate a strategy of using the body’s own repair toolkit in a targeted, safe manner. As one publication aptly noted, stem cell therapy *“without the cells”* is now within reach, and it retains the regenerative power while removing many risks. These stem cell-derived exosomes constitute a promising and possibly paradigm-shifting therapeutic for radiation-induced skin damage.

## Data Availability

Data sharing is not applicable – as no new data were generated.
